# Tumoral melanosis without metastasis: a report after three years of follow-up^☆^^☆☆^

**DOI:** 10.1016/j.abd.2020.04.016

**Published:** 2021-09-29

**Authors:** Anna Carolina Miola, Ana Claudia Cavalcante Esposito, Hamilton Ometto Stolf, Helio Amante Miot

**Affiliations:** a Faculty of Medicine, Universidade Estadual Paulista, Botucatu, SP, Brazil; b Dermatology Discipline, Universidade Estadual de Campinas, Campinas, SP, Brazil

Dear Editor,

Tumoral melanosis is a rare clinical manifestation of a completely regressed melanoma, usually represented by a pigmented lesion clinically suspected of invasive melanoma. The histopathological examination shows a dense dermal melanophage infiltrate but no atypical melanocytes.^1^ The prognosis for this unusual entity is uncertain, but metastases have been described during follow-up or even at the diagnosis.^2^

This is the report of a white female patient, aged 56 years, with no previous history of sunburns and with a dark pigmented lesion on the back, measuring 1.2 cm in diameter and showing a hypopigmented halo, which was detected during medical consultation and without a history of growth. Dermoscopy disclosed areas of peppering in the periphery, irregular edges, and a bluish-gray veil (Fig. 1). No hardened or enlarged lymph nodes were found during palpation.

**Figure 1:**
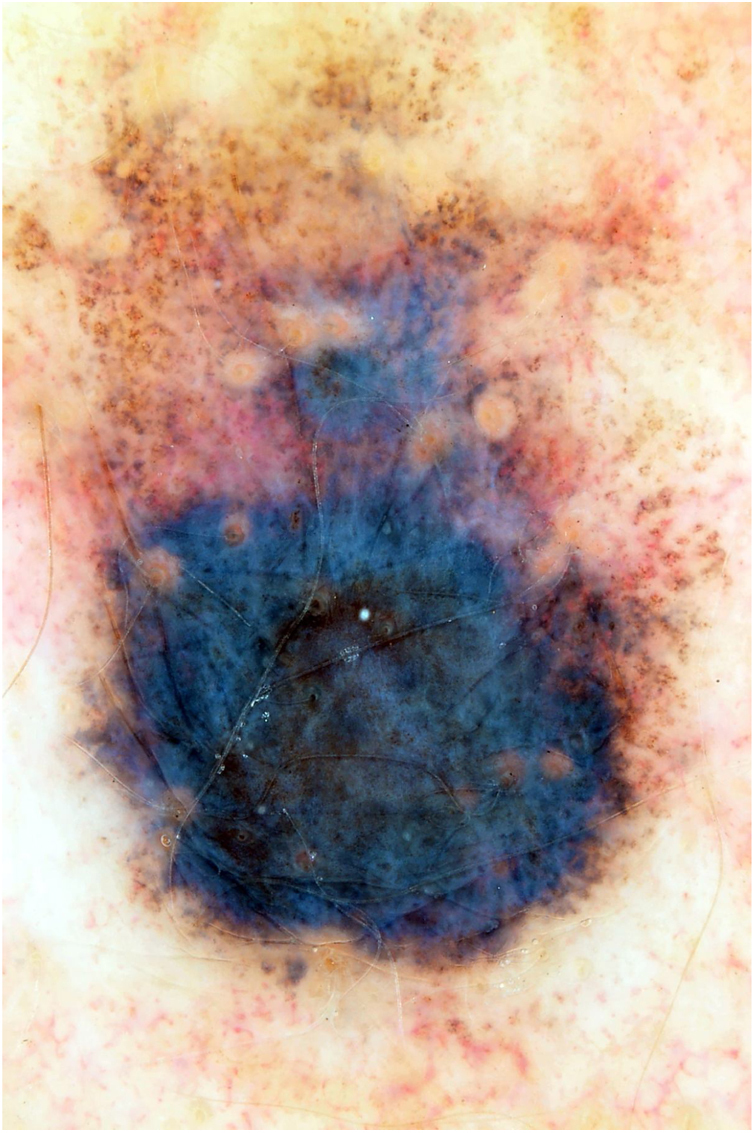
Blackish lesion, measuring 1.2 cm in diameter, with a hypopigmented halo; dermoscopy shows irregular borders and a bluish-gray veil.

An excisional biopsy with a 2-mm margin was performed, considering the hypothesis of melanoma, and the histopathological examination revealed multiple aggregates of melanophages in the reticular dermis (Clark III), better observed after counterstaining with Giemsa (Figure 2, Figure 3). The diagnosis of tumoral melanosis was established and a choice was made for enlargement with margins measuring 2 cm in diameter. Clinical examination and total body computed tomography did not disclose metastatic lesions. An abdominal and lymph node ultrasonography was performed, which did not disclose the presence of enlargement or signs of metastasis. The patient was maintained under clinical follow-up every three months, for three consecutive years, with no signs of local recurrence of the lesion or metastasis, confirmed by clinical and ultrasonographic examination.

**Figure 2:**
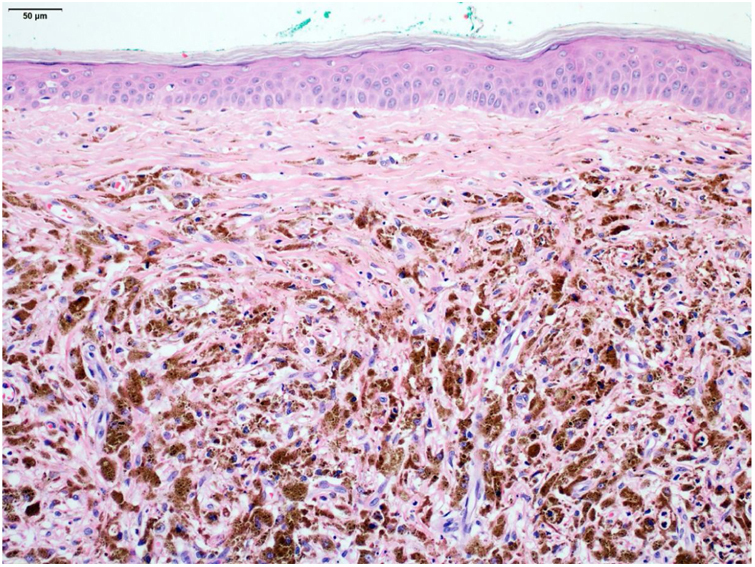
Close-up of the heavy melanophage infiltrate in the dermis (Hematoxylin & eosin, ×100).

**Figure 3:**
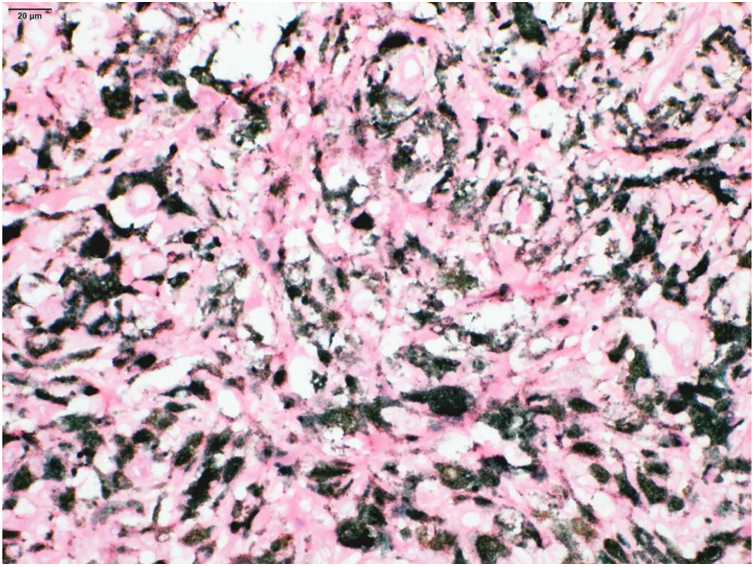
Presence of macrophages full of melanin pigment (melanophages), better seen in H&E after counterstaining with Giemsa.

Regression is a common occurrence in melanocytic neoplasias and is expected to occur in approximately 30% of cases. It usually occurs focally and seems to have little or no effect on the prognosis of an excised melanoma. However, extensive areas of regression are associated with a worse prognosis.^3^ Since tumoral melanosis represents complete regression of atypical melanocytic cells, they can also be found in lymph nodes with clinical signs of metastasis. Tumoral melanosis has also been reported following treatment of metastatic melanoma with monoclonal antibodies (e.g., dabrafenib/trametinib).^4^

Conflicting data have been reported in the literature on the prognostic effect of regression on melanoma. It is suggested that partial regression in less than 50%–75% of tumor cells does not affect prognosis, whereas complete or extensive regression above this percentage of tumor tissue is associated with metastatic disease.

The level of melanophage infiltrate is commonly correlated with the invasiveness of the previous lesion, in addition to other histopathological signs, such as solar elastosis in tumoral melanosis.^5^ In the present case, although the melanophages are located in the dermis – without contact with the epidermis, which makes it difficult to state that it was a primary lesion – it is believed that the patient had a thin melanoma that progressed into complete regression, explaining the follow-up without recurrence or metastasis for a period of three years.

The present case highlights the importance of knowing the unusual histopathology of this lesion and calls attention for the necessity of a close follow-up, even in cases with an apparent good evolution.

## Financial support

None declared.

## Authors’ contributions

Anna Carolina Miola: Approval of the final version of the manuscript; drafting and editing of the manuscript; intellectual participation in the propaedeutic and/or therapeutic conduct of the studied cases; critical review of the literature.

Ana Claudia Cavalcante Esposito: Approval of the final version of the manuscript; drafting and editing of the manuscript; critical review of the literature.

Hamilton Ometto Stolf: Approval of the final version of the manuscript; drafting and editing of the manuscript; critical review of the literature; critical review of the manuscript.

Helio Amante Miot: Approval of the final version of the manuscript; drafting and editing of the manuscript; critical review of the literature; critical review of the manuscript.

## Conflicts of interest

None declared.
